# Irisin and betatrophin responses to 9 h of passive heat exposure: Influence of age, hypertension, and type 2 diabetes

**DOI:** 10.14814/phy2.70411

**Published:** 2025-06-24

**Authors:** Joel M. Garrett, James J. McCormick, Kelli E. King, Robert D. Meade, Pierre Boulay, Ronald J. Sigal, Fergus K. O'Connor, Glen P. Kenny

**Affiliations:** ^1^ School of Health Sciences and Social Work Griffith University Southport Queensland Australia; ^2^ Human and Environmental Physiology Research Unit, School of Human Kinetics University of Ottawa Ottawa Ontario Canada; ^3^ Harvard T.H. Chan School of Public Health Harvard University Boston Massachusetts USA; ^4^ Faculty of Physical Activity Sciences University of Sherbrooke Sherbrooke Quebec Canada; ^5^ Departments of Medicine, Cardiac Sciences and Community Health Sciences, Faculties of Medicine and Kinesiology University of Calgary Calgary Alberta Canada; ^6^ Clinical Epidemiology Program Ottawa Hospital Research Institute Ottawa Ontario Canada

**Keywords:** aging, chronic disease, climate change, extreme heat, heat waves, metabolic stress

## Abstract

Age‐ and disease‐related metabolic responses to prolonged passive heat exposure are poorly understood. We evaluated serum irisin and betatrophin responses to 9 h of passive heat exposure (40°C, 9% relative humidity) in 19 young adults (19–31 years) and 37 older adults (61–78 years), including those with hypertension (HTN) and type 2 diabetes (T2D). Serum concentrations of irisin and betatrophin were assessed at baseline and at the end of the passive heat exposure using enzyme‐linked immunosorbent assays. Generalized linear models were employed to examine changes over time and across subgroups, with fold‐change computed as exponentiated coefficients. Younger adults exhibited significantly higher baseline irisin (2.81‐fold, *p* = 0.003) and betatrophin (8.15‐fold, *p* < 0.001) levels compared to older adults. Betatrophin concentrations were further reduced in older adults with HTN (0.55‐fold, *p* = 0.02) and T2D (0.55‐fold, *p* = 0.047). Despite inducing physiological strain, 9 h of passive heat exposure did not alter circulating irisin or betatrophin concentrations in any group (*p* > 0.66). While passive heat exposure alone does not trigger metabolic hormone responses, lower baseline concentrations may indicate reduced cellular heat tolerance with aging. These findings highlight potential metabolic vulnerabilities in older and heat‐sensitive populations and build on previous findings in this series.

ClinicalTrials.gov identifier: NCT04353076

## 1 INTRODUCTION

Global temperatures are rising due to climate change, leading to more frequent and severe heat waves that pose significant health risks (Amin et al., 2021; Romanello et al., 2021). Older adults are particularly vulnerable due to impaired thermoregulatory, cardiovascular, and cellular responses to heat stress (Fouillet et al., 2006; Kenny et al., 2010, 2017; Meade et al., 2020; Ndlovu & Chungag, 2024; Rey et al., 2007), placing them at an elevated risk of heat stroke (Hopp et al., 2018; Kenny et al., 2010; Meade et al., 2020), major adverse cardiovascular events (Meade et al., 2020; Wang et al., 2019), and acute kidney injury (Hopp et al., 2018; Wang et al., 2019). Recent work from our group has highlighted age‐related differences in the cellular stress response during heat exposure, including altered autophagy, apoptosis, and inflammatory pathways in peripheral blood mononuclear cells (PBMCs) (McCormick et al., 2023; Lee et al., 2024; McCormick et al., 2020). These impairments may contribute to greater susceptibility to heat‐related illness, weakened immune function, and reduced capacity for cellular repair in older adults. However, changes in circulating proteins associated with metabolic regulation during prolonged heat stress remain underexplored.

Irisin and betatrophin warrant attention in prolonged heat exposure settings because they serve crucial roles in regulating metabolic homeostasis and cellular stress responses. Irisin, produced by the cleavage of fibronectin type III domain‐containing protein 5 (FNDC5), is released in response to acute stressors, such as exercise, and exerts cytoprotective effects by mitigating oxidative stress and cellular damage (Guo et al., 2024; Liu et al., 2022; Wang et al., 2020). Betatrophin, primarily secreted by the liver, modulates lipid metabolism and glucose balance, and has also been implicated in pancreatic β‐cell proliferation (Enteshary et al., 2019; Fu, Berhane, et al., 2014; Rejeki et al., 2022; Tseng et al., 2014). Emerging evidence indicates that heat stress can alter circulating levels of irisin, as demonstrated by elevated irisin following hot water immersion in young females (Park et al., 2021) and in younger adults during prolonged exercise in the heat (McCormick, King, Notley, Fujii, Boulay, Sigal, & Kenny, 2022). The latter report also showed that irisin responses to exercise heat stress were blunted in older compared to younger adults (McCormick, King, Notley, Fujii, Boulay, Sigal, & Kenny, 2022). Although the influence of acute heat stress on betatrophin remains unexplored, its known metabolic functions and irisin's impact on betatrophin's expression and secretion (Tseng et al., 2014) suggest it may be similarly responsive. These hormones may help clarify whether disruptions in glucose and lipid regulation under heat stress contribute to broader health risks associated with prolonged heat exposure.

Despite the growing interest in irisin and betatrophin as mediators of metabolic health, their response to acute physiological stress, including heat stress, remains unclear. This gap in knowledge is particularly relevant given that chronic health conditions such as hypertension (HTN) and type 2 diabetes (T2D) are associated with heightened oxidative stress (Pouvreau et al., 2018), potentially increasing reliance on irisin's cytoprotective effects to mitigate oxidative damage during acute stressors. Older adults, who already exhibit greater oxidative stress and inflammatory responses during heat exposure due to impaired thermoregulatory function (McCormick et al., 2023), may be even more susceptible to these metabolic disruptions. Understanding whether irisin and betatrophin responses are further blunted in older adults with HTN or T2D could provide valuable insight into the metabolic vulnerabilities that contribute to heat‐related morbidity and mortality.

We investigated whether serum concentrations of irisin and betatrophin change following 9 h of passive heat exposure in young and older adults, building on previous findings in this series that highlighted impairments in thermoregulation and cellular stress responses in older adults (McCormick et al., 2023; Lee et al., 2024; Meade et al., 2023). Given the roles of these hormones in metabolic regulation, we hypothesized that both would increase post‐heat exposure, with blunted responses in older adults, particularly those with HTN or T2D.

## 2 METHODS

### 2.1 Participants

The total sample analyzed in this report includes 19 young adults (19–31 years) and 37 older adults (61–78 years) (Table 1). Older participants were further categorized into subgroups based on their health status: without HTN or T2D (*n* = 17), with HTN (*n* = 10), and with T2D (*n* = 10). Five participants with T2D also had HTN, resulting in 15 participants with HTN. Full participant characteristics included within this study and duration of disease states (if applicable) are presented in Table 1. Participants were recruited from the Ottawa, Canada area and provided written informed consent prior to taking part in the study. A detailed account of the inclusion and exclusion criteria, participant screening including a full list of current medications, along with the thermoregulatory and hemodynamic responses to prolonged heat stress is presented in the first report of this series by Meade et al. (2023); only details relevant to the current study are repeated here.

**TABLE 1:** Participant characteristics adapted from Meade et al. (2023).

	Young adults (*n* = 19)	Older adults			
		All older adults (*n* = 37)	Without T2D or HTN (*n* = 17)	With T2D^d^ (*n* = 10)	With HTN^e^ (*n* = 15)
Age, years	24 (21–28)	70 (68–73)	71 (70–74)	72 (68–72)	68 (67–73)
Sex					
Female	10 (53%)	10 (27%)	6 (35%)	2 (20%)	4 (27%)
Male	9 (47%)	27 (73%)	11 (65%)	8 (80%)	11 (73%)
Height (cm)	168 (164–172)	171 (166–177)	170 (164–175)	170 (166–178)	171 (166–176)
Body mass (kg)	64.3 (58.1–82.2)	75.9 (69.5–84.0)	71.5 (66.0–77.5)	76 (70.8–82.3)	79 (70.5–84.3)
Body mass index, kg/m^2^ ^a^	24.1 (22.2–25.8)	25.9 (24.2–28.3)	25.4 (21.4–27.3)	26.8 (25.6–28)	26.8 (24.6–29.0)
Body surface area m^2^ ^b^	1.8 (1.6–2.0)	1.9 (1.8–2.0)	1.8 (1.7–2.0)	1.9 (1.8–1.9)	1.9 (1.8–2.0)
Systolic blood pressure (mm Hg)	114 (110–120)	127 (119–135)	125 (120–135)	121 (113–131)	132 (118–138)
Diastolic blood pressure (mm Hg)	73 (68–77)	74 (68–79)	75 (69–79)	68 (58–73)	73 (66–78)
Physical activity, min/week^c^	180 (150–225)	200 (138–300)	120 (85–311)	200 (180–438)	195 (173–255)
Types of physical activity					
Walking	14 (74%)	29 (78%)	14 (82%)	7 (70%)	12 (80%)
Jogging, biking, and swimming	14 (74%)	19 (51%)	9 (53%)	5 (50%)	7 (47%)
Aerobics and floor exercises	2 (11%)	8 (22%)	7 (41%)	0 (0%)	1 (7%)
Organized sports	14 (74%)	7 (19%)	2 (12%)	3 (30%)	4 (27%)
Prescription medications					
Yes	6 (32%)	31 (84%)	11 (65%)	10 (100%)	15 (100%)
No	13 (68%)	6 (16%)	6 (35%)	0 (0%)	0 (0%)

*Note*: Values are median (interquartile range) or number of participants (%).

Abbreviations: HTN, hypertension; T2D, type 2 diabetes.

^a^ Body mass index calculated as weight in kilograms divided by the square of the height in meters.

^b^ Body surface area calculated according to the equation by Du Bois & Du Bois, (1989).

^c^ Participant self‐reported physical activity level determined via the Canadian Society for Exercise Physiology Get Active Questionnaire (GAQ) (Tremblay et al., 2011) while the general types of physical activity performed were assessed using the Kohl Physical Activity Questionnaire (Kohl et al., 1988).

^d^ Length of time since diagnosis of type 2 diabetes (T2D) ranged from 4 to 20 years. Five participants with T2D also had hypertension (HTN). Glycated hemoglobin (hemoglobin A1C) measured in a subset of participants (*n* = 5) was 6.0%–8.2% (*n* = 4 had hemoglobin A1C < 7%).

^e^ Length of time since diagnosis of HTN ranged from 4 to 40 years.

### 2.2 Experimental protocol

A detailed account of the inclusion and exclusion criteria, participant screening including a full list of current medications, along with the thermoregulatory and hemodynamic responses to prolonged heat stress is presented in the first report of this series by Meade et al. (2023) (ClinicalTrials.gov identifier: NCT04353076); only details relevant to the current study are included here. Participants completed a 9‐h passive heat exposure trial in a climate‐controlled chamber maintained at 40°C with 9% relative humidity. These environmental conditions were selected to simulate maximum temperatures recorded during North American heat waves (McCormick et al., 2023; Lee et al., 2024; Meade et al., 2023).

Upon arrival, participants changed into lightweight summer clothing (sandals, shorts, and a light top) and, after instrumentation and baseline measurements, entered the chamber. The trial consisted of three phases: during hours 1–3 and 7–9, participants were seated within the Snellen air calorimeter to assess whole‐body heat exchange and storage. During hours 4–6, participants remained seated outside the calorimeter while still exposed to the hot, dry conditions. Water was available ad libitum throughout this time period, and participants consumed a light, self‐provided lunch during the mid‐exposure phase. Body mass was measured before and after heat exposure to assess fluid loss.

### 2.3 Blood sampling and biochemical analysis

Venous blood samples were collected at two time points: baseline (prior to heat exposure) and at the end of the 9‐h heat exposure (while participants were still in the heat). Blood samples were drawn from an antecubital vein after participants had been seated for at least 45 min to ensure a resting state. Blood was collected into serum separator tubes and allowed to clot for approximately 15 min before centrifugation at 1380 × G for 10 min. Serum was aliquoted and stored at −80°C for subsequent analysis.

Irisin and betatrophin concentrations were measured using commercially available enzyme‐linked immunosorbent assay (ELISA) kits (irisin: DY9420, Bio‐Techne, Oakville, ON, Canada; betatrophin: MBS166727, MyBioSource, San Diego, CA, USA), following the manufacturer's instructions at a 1:5 dilution of sample:reagent diluent. All samples were assayed in duplicate to ensure reliability, with an intra‐assay coefficient of variation of <5%. Concentrations were reported as geometric means, and fold changes were calculated by comparing post‐exposure values to baseline.

### 2.4 Statistical analysis

All statistical analyses were conducted using R statistical software (version 4.4.0). Data distributions for normality were visually assessed through histograms and Q‐Q plots. Both serum irisin and betatrophin concentrations were non‐normally distributed at baseline and end‐exposure. Log transformation did not fully normalize the data. Therefore, generalized linear models (GLMs) with a Gamma distribution and log link function were employed for analysis (Wilson et al., 1996). Since the Gamma distribution requires strictly positive values, a small constant (1e‐4) was added to the baseline‐to‐end‐exposure values, which included both positive and negative changes, to shift all values above zero while preserving relative differences. For all GLMs, results were reported as fold change and percentage change (%), with 95% confidence intervals (CIs). Fold change was computed as exponentiated coefficients (Exp(β)), while percentage change was calculated as (Exp(β) − 1) × 100. Statistical significance was set at *p* < 0.05, and 95% CIs were used to assess the precision of estimates. In addition to the GLM results and fold‐change estimates, we calculated the raw median differences or changes. All GLMs were checked for homoscedasticity and model fit. Residuals were examined for deviations from normality, and model dispersion parameters were inspected to ensure proper fit.

### 2.5 Baseline differences in irisin and betatrophin

To evaluate differences in baseline irisin levels between young and older adults, we first ran a GLM to compare the two age groups. Given the presence of HTN and T2D in the older adult cohort, we then adjusted for these variables in the model. This model accounted for potential confounding effects of HTN and T2D when comparing irisin levels between age groups. However, to further isolate age‐related effects from HTN and T2D‐related differences, a secondary analysis was conducted, restricting the older adult cohort to those without HTN or T2D and comparing them to the young adult group. Lastly, to investigate whether HTN and T2D independently influenced baseline irisin concentrations within older adults, a separate model was run including only the older adult cohort.

### 2.6 Baseline to end‐exposure changes in irisin and betatrophin

To evaluate baseline to end‐exposure changes in irisin and betatrophin, a GLM was fitted including time (baseline to end‐exposure) and age group (young vs. older adults) to assess whether changes over time differed by age. Given the presence of HTN and T2D in the older adult cohort, these conditions were again included as covariates to account for potential confounding effects. A secondary analysis was conducted to further isolate age‐related effects by restricting the older adult cohort to those without HTN or T2D and comparing their baseline to end‐exposure changes with young adults. Lastly, within the older adult cohort, additional GLMs examined whether HTN and T2D influenced baseline to end‐exposure changes, including interaction terms to assess their potential modifying effects.

## 3 RESULTS

### 3.1 Body temperature and fluid regulation responses

Analyses for body core temperature and hemodynamic regulation are presented within our primary outcome paper (Meade et al., 2023) and were not part of this analysis. Here, we present a summary of the body core temperature data to provide context for the reader. The increase in body core temperature over the 9‐h exposure was 0.3°C (95% confidence interval: 0.1, 0.4) higher in older adults compared to younger adults (older 0.9 (0.3)°C vs. young: 0.6 (0.3)°C, *p* = 0.007). No between‐group differences in mean body temperature occurred (*p* = 0.310). However, cumulative thermal strain, indexed via the area under the curve for both body core temperature and mean body temperature, was significantly greater in older adults (*p* < 0.001 and *p* = 0.012, respectively). The decline in plasma volume was 4.0% greater in older adults (3.9% (standard deviation 3.9%) vs. young: 0.1% (5.1%); *p* = 0.002). Conversely, no significant difference was observed in body weight change between groups (young: 1.9% (0.9%) vs. older: 1.9% (0.9%); difference: 0.0% (−0.5, 0.5%), *p* = 0.971).

### 3.2 Irisin

#### 3.2.1 Differences in baseline levels

**FIGURE 1:**
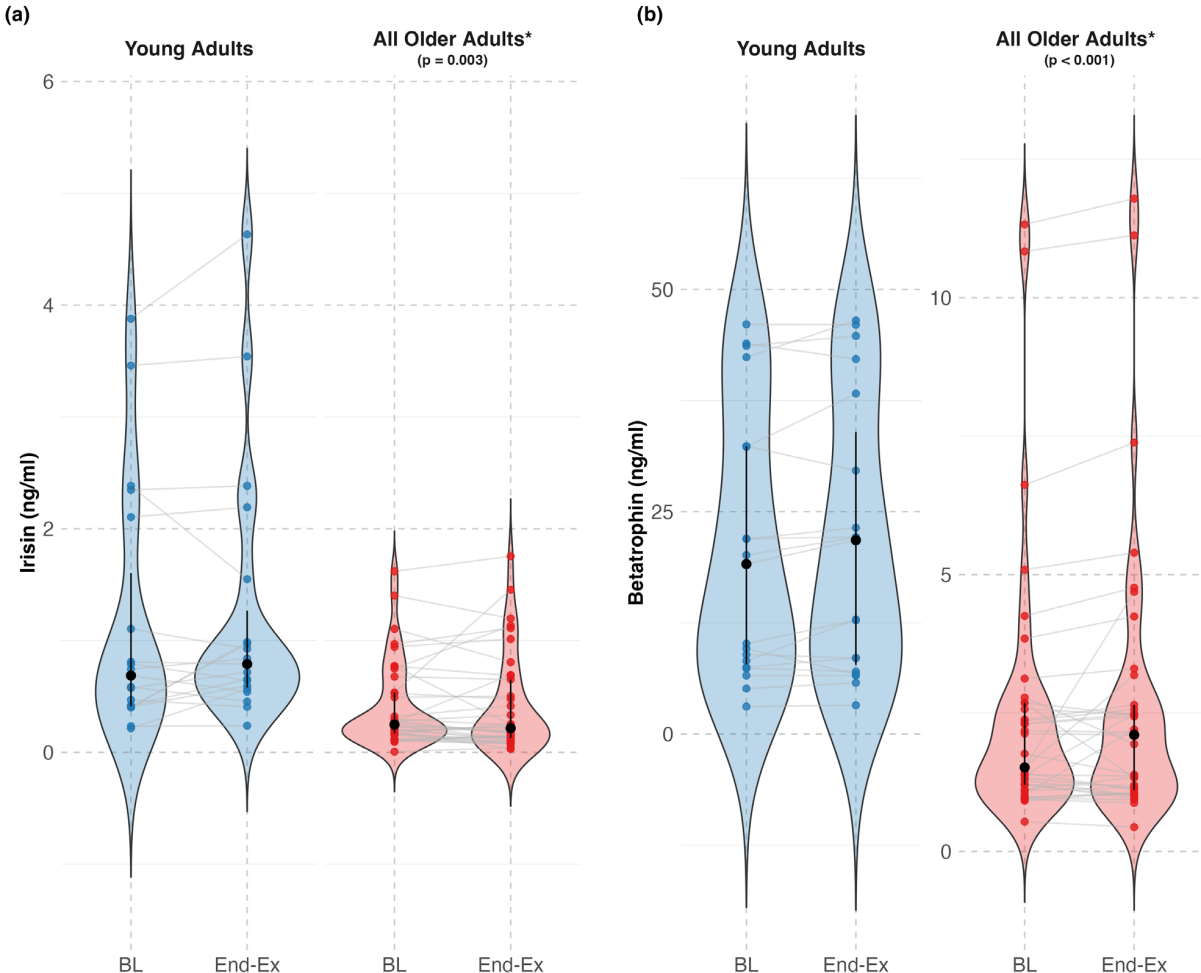
Baseline and end‐exposure irisin and betatrophin concentrations for young and older adults. Panel a: Baseline (BL) and end‐exposure (End‐Ex) irisin concentrations in young adults (blue) and older adults (red). Panel b: Baseline and end‐exposure betatrophin concentrations in young adults (blue) and older adults (red). Black circles with error bars indicate the median and interquartile range (IQR) for each group. Violin plots illustrate the distribution of hormone concentrations at each time point. Individual data points represent participant values, with gray lines connecting baseline and end‐exposure measurements. *Indicates a significant difference from young adults at baseline.

**FIGURE 2:**
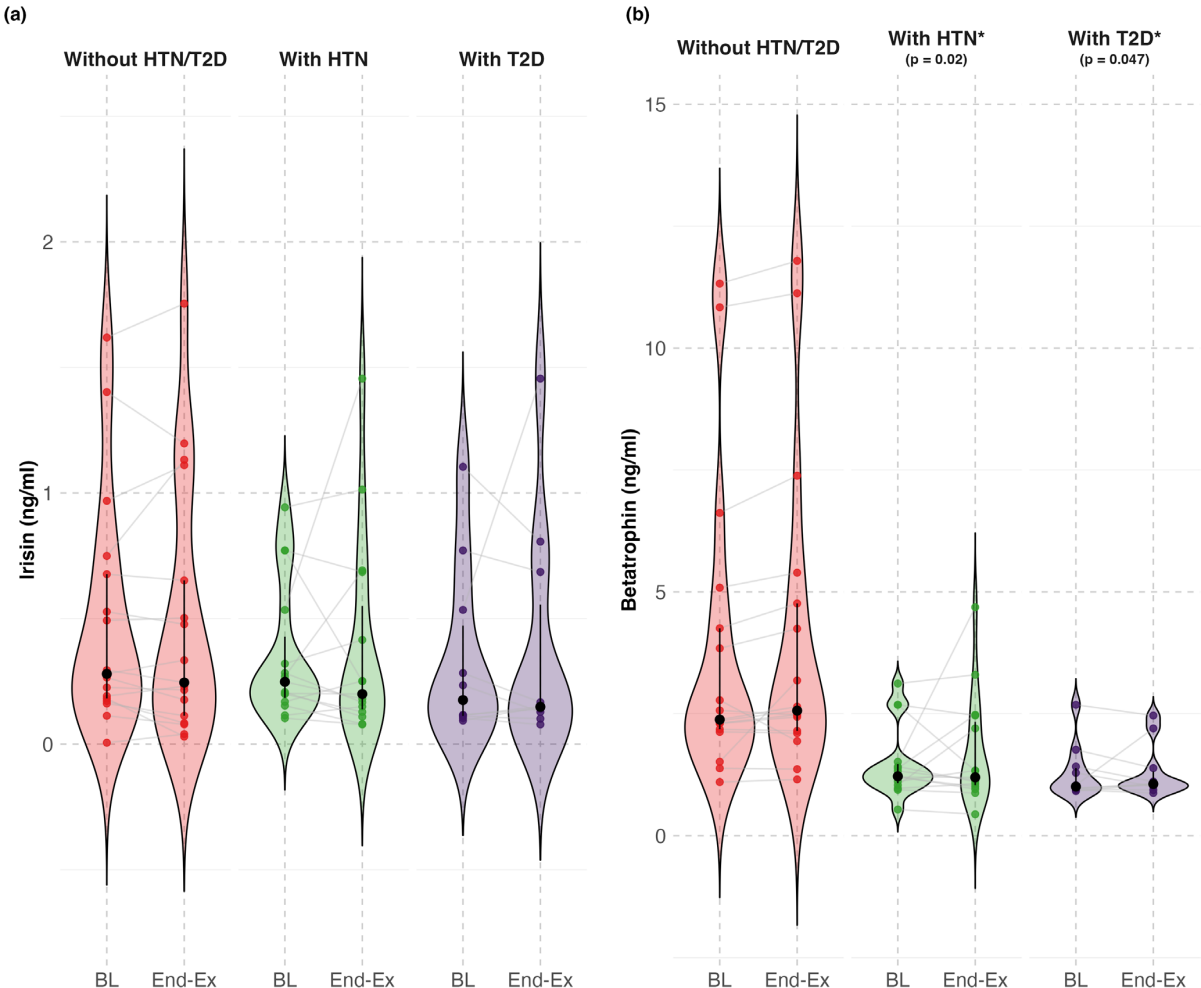
Baseline and end‐exposure irisin and betatrophin concentrations across older adult comparison groups (older adults without T2D or HTN, with HTN, and with T2D). Panel a: Baseline (BL) and end‐exposure (End‐Ex) irisin concentrations in older adults without hypertension (HTN) or type 2 diabetes (T2D) (red), with HTN (green) and with T2D (purple). Panel b: Baseline and post‐exposure betatrophin concentrations in older adults without HTN or T2D (red), with HTN (green) and with T2D (purple). Black circles with error bars indicate the median and interquartile range (IQR) for each group. Violin plots illustrate the distribution of hormone concentrations at each time point. Individual data points represent participant values, with gray lines connecting baseline and end‐exposure measurements. Five participants with T2D also had hypertension and are reflected in both the with HTN and T2D groups. *Indicates a significant difference from older adults without HTN or T2D at baseline.

Young adults had 2.81‐fold higher irisin levels than older adults (raw median difference 0.44 ng/mL, 181% higher, 95% CI: 68%–381%, *p* = 0.003) (Figure 1). When adjusting for HTN and T2D, the effect of age remained significant, with young adults having 2.47‐fold higher irisin levels (147% higher, 95% CI: 37%–348%, *p* = 0.004). To further isolate age‐related effects from chronic disease‐related differences, a secondary analysis compared young adults to older adults without HTN or T2D. In this model, young adults still exhibited 2.37‐fold higher irisin levels (137% higher, 95% CI: 26%–342%, *p* = 0.01), confirming that age differences in irisin levels persist independent of HTN and T2D. Within the older adult cohort, neither HTN (raw median difference 0.05 ng/mL, 20% higher, 95% CI: −56% to 53%, *p* = 0.49) nor T2D (raw median difference 0.10 ng/mL, 17% higher, 95% CI: −57% to 74%, *p* = 0.60) were significantly associated with baseline irisin levels (Figure 2).

#### 3.2.2 Baseline to end‐exposure changes

There was no significant main effect of time, indicating that irisin levels remained stable across exposure for both age groups (3% change, 95% CI: −34% to +61%, *p* = 0.91, raw median change in young adults was 0.10 ng/mL and –0.03 ng/mL in all older adults). The time × age group interaction was also not significant (5% change, 95% CI: −52% to +125%, *p* = 0.91), suggesting no difference in irisin responses between young and older adults. To further evaluate age‐related differences independent of HTN or T2D, a secondary analysis compared young adults to older adults without HTN or T2D. Similar to the primary model, there was no significant effect of time (1% change, 95% CI: −48% to 94%, *p* = 0.99) or a time × age group interaction (7% change, 95% CI: −57% to 162%, *p* = 0.89). Within the older adult group, there was no significant change in irisin from baseline to end exposure (−5% change, 95% CI: −41% to 77%, *p* = 0.88). In older adults without HTN or T2D, there was a raw median change of −0.03 ng/mL; in those with HTN, the raw median change was −0.003 ng/mL; and in those with T2D, it was −0.03 ng/mL. Neither HTN (13% change, 95% CI: −57% to 200%, *p* = 0.80) nor T2D (11% change, 95% CI: −62% to 227%, *p* = 0.85) modified irisin responses to heat exposure.

### 3.3 Betatrophin

#### 3.3.1 Differences in baseline levels

Young adults had 8.15‐fold higher betatrophin levels (raw median difference 17.59 ng/mL, 715% higher, 95% CI: 403%–1260%, *p* < 0.001) (Figure 1). When adjusting for HTN and T2D, the effect of age remained significant, with young adults having 5.97‐fold higher betatrophin levels (497% higher, 95% CI: 277%–847%, *p* < 0.001). To further isolate age‐related effects from HTN and T2D‐related differences, a secondary analysis compared young adults to older adults without HTN or T2D. In this model, young adults still exhibited 5.38‐fold higher betatrophin levels (438% higher, 95% CI: 224%–788%, *p* < 0.001), confirming that age‐related differences persist independent of HTN and T2D. Unlike irisin, betatrophin levels were 0.55‐fold lower in older adults with HTN or T2D compared to older adults without either condition (HTN: raw median difference −1.11 ng/mL, 55% lower, 95% CI: −67% to −8%, *p* = 0.02; T2D: raw median difference −1.37 ng/mL, 55% lower, 95% CI: −68% to −1%, *p* = 0.047) (Figure 2). These findings suggest that both HTN and T2D negatively impact baseline betatrophin levels.

#### 3.3.2 Baseline to end‐exposure changes in betatrophin

There was no significant main effect of time, indicating that betatrophin levels remained stable across exposure for both age groups (8% change, 95% CI: −23% to 51%, *p* = 0.66, raw median change in young adults was 2.69 ng/mL and 0.59 ng/mL in all older adults). The time × age group interaction was also not significant (−3% change, 95% CI: −45% to 72%, *p* = 0.92), suggesting no difference in betatrophin responses between young and older adults. To further evaluate age‐related differences independent of HTN or T2D, a secondary analysis compared young adults to older adults without HTN or T2D. Similar to the primary model, there was no significant effect of time (7% change, 95% CI: −36% to 78%, *p* = 0.81) or a time × age group interaction (−2% change, 95% CI: −53% to 99%, *p* = 0.96). Within the older adult group, there was no significant change in betatrophin from baseline to end‐exposure (6% change, 95% CI: −33% to 67%, *p* = 0.82). In older adults without HTN or T2D, there was a raw median change of 0.18 ng/mL; in those with HTN, the raw median change was −0.008 ng/mL, and in those with T2D, it was 0.06 ng/mL. Neither HTN (12% change, 95% CI: −47% to 136%, *p* = 0.75) nor T2D (−9% change, 95% CI: −60% to 106%, *p* = 0.80) modified betatrophin responses to 9 h of heat exposure.

## 4 DISCUSSION

This study investigated the effects of a 9‐h exposure to extreme heat on the metabolic hormones irisin and betatrophin in young and older adults, with and without common chronic health conditions (HTN and T2D). Our findings complement the broader physiological insights reported previously (McCormick et al., 2023; Lee et al., 2024; Meade et al., 2023) by highlighting that while inflammatory and cellular stress hormones respond dynamically to prolonged exposure to extreme heat, these metabolic hormones remain stable across different age and health subgroups. This stability underscores the distinct roles of inflammatory versus metabolic pathways in the heat stress response.

### 4.1 Differences in baseline levels

Our study found that young adults had significantly higher baseline levels of irisin and betatrophin compared to older adults. These results align with our previous work showing that irisin levels are lower in older adults, even in temperate environments (McCormick, King, Notley, Fujii, Boulay, Sigal, & Kenny, 2022). The lower irisin levels with aging may reflect a decrease in muscle mass (Chang et al., 2017; Planella‐Farrugia et al., 2019) and metabolic function (Park et al., 2013; Polyzos et al., 2018). In line with previous work (McCormick, King, Notley, Fujii, Boulay, Sigal, Amano, & Kenny, 2022), the fact that irisin levels were not significantly different between older adults with and without HTN or T2D suggests that age‐related declines in irisin may be independent of metabolic disease status.

In contrast, betatrophin levels were significantly lower in older adults with HTN or T2D compared to those without these conditions, which could suggest a stronger link between betatrophin regulation and metabolic health. However, betatrophin concentrations in patients with T2D remain conflicting. Gómez‐Ambrosi et al. (2014) showed that betatrophin levels were lower in patients with T2D, while Chen et al. (2015) demonstrated higher betatrophin levels in T2D patients. These differences may be attributed to methodological differences, participant demographics, or length and/or severity of the chronic condition diagnosis (Fu, Abou‐Samra, & Zhang, 2014). It has also been speculated that hyperinsulinism and/or insulin resistance might result in decreased betatrophin levels as a negative feedback mechanism, leading to increased insulin secretion under conditions of typical glucose homeostasis and insulin sensitivity (Tuhan et al., 2016). Nevertheless, changes from typical betatrophin concentration would suggest diminished metabolic flexibility in situations of increased metabolic stress.

### 4.2 Baseline to end‐exposure changes

Despite the well‐established role of irisin in thermoregulation and energy metabolism (Flori et al., 2021; Shen et al., 2022), this study found no significant changes in circulating irisin levels following 9 h of exposure to extreme heat in either young or older adults. This contrasts with previous research demonstrating increases in irisin following exercise‐induced thermogenesis (Daskalopoulou et al., 2014; Tsuchiya et al., 2014, 2015), suggesting that muscular contractions may be a necessary stimulus for increased irisin release. The absence of significant baseline to end‐exposure changes indicates that passive heat exposure alone may not be sufficient to alter irisin secretion. As irisin has a positive impact on betatrophin expression and secretion (Tseng et al., 2014), it is unsurprising that a similar response in betatrophin levels was observed.

These findings highlight a fundamental difference between exercise‐induced and heat‐induced stress responses. While previous research suggests that exercise promotes irisin secretion, findings have been inconsistent (Fox et al., 2018; Guo et al., 2024; Liu et al., 2022; Tsuchiya et al., 2015). For example, prolonged moderate‐intensity exercise (3 h at ~40% of maximal aerobic capacity (VO_2_max)) in a non‐heat stress environment from our group has not been shown to induce an increase in irisin production (McCormick, King, Notley, Fujii, Boulay, Sigal, Amano, & Kenny, 2022; McCormick, King, Notley, Fujii, Boulay, Sigal, & Kenny, 2022). However, irisin concentrations were shown to increase in young and older adult groups with and without chronic health conditions during prolonged moderate‐intensity exercise in the heat (McCormick, King, Notley, Fujii, Boulay, Sigal, Amano, & Kenny, 2022; McCormick, King, Notley, Fujii, Boulay, Sigal, & Kenny, 2022). This suggests that a metabolically induced stress, rather than heat exposure alone, is a primary driver of irisin secretion. A daylong exposure to extreme heat, while capable of inducing physiological strain (Meade et al., 2023), appears insufficient to elicit the metabolic stress required to upregulate irisin and betatrophin levels.

This distinction is particularly relevant for heat‐vulnerable populations. While prolonged heat exposure has been shown to negatively impact inflammatory responses, gastrointestinal barrier integrity, and body core temperature regulation, particularly in older adults and those with HTN and T2D (McCormick et al., 2023; Lee et al., 2024; Meade et al., 2023), the stability of irisin and betatrophin suggests that these metabolic hormones may play a more sustained, long‐term role in baseline metabolic regulation rather than responding acutely to heat stress. Unlike exercise, which imposes a significant metabolic demand due to increased muscle contractions and substrate utilization (Amin et al., 2021), passive heat exposure primarily elicits thermoregulatory responses such as sweating and cutaneous vasodilation, which have a relatively low energy cost (Kenny & Flouris, 2014). However, the cumulative physiological strain from prolonged or repeated heat exposure may still influence systemic metabolic regulation over time. Further studies are required to assess whether these responses remain intact over successive days of exposure to conditions typical of hot weather or extreme heat events.

### 4.3 Limitations

This study has some limitations that should be considered when interpreting the findings. Sample size calculations were conducted for the primary outcomes reported previously (Meade et al., 2023). However, the present sub‐analyses were exploratory in nature and may be underpowered to detect subtle differences, limiting the generalizability of these findings. Larger, more diverse cohorts will be needed to confirm these exploratory observations and further investigate potential interactions between age, sex, and chronic health conditions, such as HTN and T2D. Additionally, our study participants with HTN and T2D were well‐managed, meaning their metabolic and vascular responses to heat exposure may differ from individuals with poorly controlled conditions. Future studies should examine whether disease severity or medication use influences metabolic hormone responses to heat stress. Second, the passive heat exposure protocol used in this study does not fully replicate real‐world heat wave conditions where exposure to extreme heat can occur over multiple days. Additionally, it did not consider the influence of activities of daily living, which would exacerbate the increase in physiological strain while inducing an elevated state of metabolic stress. Since exercise is a known stimulus for irisin release (Flori et al., 2021; Shen et al., 2022), future studies should further investigate the combined effects of heat and exercise to determine whether this increased metabolic stress plays a role in the release of irisin and betatrophin during environmental heat stress. Another key limitation is that biomarker measurements were only taken at baseline and at the end of the 9‐h exposure, providing a snapshot of metabolic responses rather than a continuous assessment of changes over time. It remains unclear whether transient fluctuations in irisin and betatrophin levels may have occurred during the 9‐h exposure or whether delayed responses would emerge in the hours or days following heat stress. Future studies incorporating repeated sampling throughout exposure and during recovery could help determine the temporal dynamics of these metabolic hormones. Lastly, it is unknown whether current medications may have an impact on circulating biomarker levels. For instance, metformin (the only drug taken by each of the the individuals with T2D in the present study) may stimulate irisin release; however, this has only been demonstrated in C2C12 myoblasts and has yet to be evaluated in humans (Seong et al., 2025). Further, the effects of metformin use on betatrophin remain unclear (Espes et al., 2014). Therefore, future investigations are required to determine the impact of common medication effects on irisin and betatrophin production.

## 5 CONCLUSION

This study examined the effects of prolonged passive heat exposure on the metabolic hormones irisin and betatrophin, considering age, HTN, and T2D. Our findings suggest that while heat exposure induces physiological strain, it does not significantly alter these hormones, distinguishing heat‐induced from exercise‐induced metabolic responses. Baseline irisin levels declined with age, independent of HTN and T2D status, while betatrophin was lower in older adults with HTN and T2D, reinforcing its link to metabolic health. Although no significant heat‐induced changes were observed, lower baseline levels may contribute to reduced cellular heat tolerance with age. These results highlight potential metabolic vulnerabilities in older and heat‐sensitive populations. Targeted interventions, particularly exercise‐based strategies, may be necessary to enhance metabolic resilience. Future research should explore whether repeated heat exposures or combined heat and exercise interventions elicit more pronounced metabolic adaptations, helping to mitigate heat‐related risks in at‐risk groups.

## AUTHOR CONTRIBUTIONS

GPK, RDM, and RJS designed the trial, conceived the research question, and acquired funding. JJM, RDM, and KEK collected data. JMG and JJM analyzed data. JMG prepared tables and figures. JMG drafted the manuscript. All authors revised the manuscript, approved the final version, and agree to be accountable for all aspects of the work. All persons designated as authors meet the International Committee of Medical Journal Editors (ICMJE) criteria for authorship. All authors had full access to and accept responsibility for the data presented in this report.

## FUNDING INFORMATION

This research was funded by the Canadian Institutes for Health Research (CIHR) Research Grant (no. 399434) and Health Canada (contract no. 4500387992). The funders had no role in trial design, collection, analysis, or interpretation of data, or in manuscript development. No authors received direct compensation related to the development of this article. JJM, KEK, RDM, and FKO were supported by the Human and Environmental Physiology Research Unit. RDM was supported by a CIHR Postdoctoral Fellowship and the Human and Environmental Physiology Research Unit. KEK was funded by a University of Ottawa International Doctoral Scholarship. GPK was supported by a University of Ottawa Research Chair.

## CONFLICT OF INTEREST STATEMENT

No conflicts of interest, financial or otherwise, are declared by the authors.

## ETHICS STATEMENT

The study was approved by the University of Ottawa Health Sciences and Science Research Ethics Board (H‐11‐18‐1186) and conducted in accordance with the Declaration of Helsinki.

## Data Availability

De‐identified participant data are available from the corresponding author (gkenny@uottawa.ca) upon reasonable request and signed access agreement.
